# Rosuvastatin ameliorates obesity-associated insulin resistance in high-fat diet-fed mice by modulating the gut microbiota and gut metabolites

**DOI:** 10.3389/fcimb.2025.1593581

**Published:** 2025-06-30

**Authors:** Chao Yao, Xin Xue, Yunxi Jia, Min Li, Lu Zhang, Hong Yuan, Huiting Xue, Ruiping Hu

**Affiliations:** College of Basic Medicine, Inner Mongolia Medical University, Hohhot, China

**Keywords:** insulin resistance, rosuvastatin, metagenome, metabolome, butyric acid

## Abstract

**Introduction:**

Insulin resistance (IR) underlies metabolic diseases such as obesity and diabetes. Statins are lipid-lowering drugs that have also been studied to improve insulin resistance, but the mechanism is not well understood. Metagenomics and metabolomics were used to analyze the main species and metabolic pathways involved in intestinal microbes while improving insulin resistance in mice with rosuvastatin in this study.

**Methods:**

C57BL/6J male mice fed a high-fat diet were used to establish the insulin resistance (IR) mouse model. Rosuvastatin (RSV) was then administered for 8 weeks. Metagenomics and metabolomics were utilized to analyze the microbial composition and short chain fatty acid metabolites in intestinal feces of mice.

**Results:**

It was observed that insulin-resistant mice showed significant improvement in insulin resistance following treatment with RSV. In comparison to the HFD group, specific bacterial strains were significantly increased, and the levels of butyric acid, caproic acid, and isovaleric acid among the short-chain fatty acids were notably elevated in the RSV group. Through KEGG enrichment analysis, 19 dominant strains and 15 key enzymes involved in butyric acid metabolism were identified.

**Conclusions:**

The results suggested that IR mice might enhance insulin sensitivity by promoting butyric acid synthesis via intestinal microbes following RSV treatment.

## 1 Introduction

With the improvement of people’s living standards, the incidence of metabolic diseases such as obesity, diabetes and metabolic syndrome is gradually on the rise, and insulin resistance plays an important role in the occurrence and development of these diseases (Boden and Merali, 2011). Insulin resistance refers to the decreased sensitivity of insulin target cells (such as skeletal muscle cells, liver cells, fat cells, etc.) to insulin, that is, the reduced efficiency of glucose uptake and utilization, resulting in abnormal glucose tolerance, etc (Lee et al., 2022).

The human gut hosts a highly diverse microbial community essential for health. The genes of the gut microbiome are known as the “second genome” of humans (Komaroff, 2018; Yang et al., 2024). Many studies have shown that gut microbes and their metabolites play an important role in insulin resistance or diabetes (Liu et al., 2020; Guo et al., 2022; Sankararaman et al., 2023). Alterations in the composition of the host gut microbiota can result in modifications to its metabolic byproducts. Among these metabolites, short-chain fatty acids (SCFAs) have been demonstrated to be associated with the pathogenesis of metabolic disorders, including insulin resistance (Agus et al., 2021). SCFAs play a significant role in the metabolism of substances and are crucial for the functioning of various organs and tissues, including the intestine, brain, bone, adipose tissue and pancreatic islets (Mukhopadhya and Louis, 2025). Additionally, SCFAs influence the metabolism of carbohydrates and lipids, thereby regulating energy metabolism (Perry et al., 2016) and contributing to the maintenance of the body’s energy homeostasis. These properties suggest that SCFAs may have potential therapeutic applications in the management of obesity and diabetes (De Vadder et al., 2014; Zhao et al., 2018; Sanna et al., 2019).

The gut microbiota is influenced by a range of factors, including host genetics, diet, lifestyle, environment and medications (Yatsunenko et al., 2012; Goodrich et al., 2014; Gaćeša et al., 2022). Rosuvastatin, which belongs to the statin family, is frequently prescribed to manage dyslipidemia. It functions by inhibiting the endogenous synthesis of cholesterol, specifically through the competitive inhibition of the enzyme 3-hydroxy-3-methylglutaryl-CoA reductase (HMG-CoA reductase) (Cortese et al., 2016). Early animal studies indicated that rosuvastatin significantly improved insulin resistance in mice, though the underlying mechanism remains unclear. Therefore, this study aims to analyze the primary species and metabolic pathways of gut microbes involved in the improvement of insulin resistance in mice treated with rosuvastatin, utilizing metagenomics and metabolomics approaches.

## 2 Materials and methods

### 2.1 Animals and reagents

With the approval of the Ethics Committee of Inner Mongolia Medical University and in accordance with the guiding principles of the Experimental Animal Care and Use Guide of Inner Mongolia Medical University, 30 5-week-old C57BL/6J male mice were purchased from Beijing Sibeifu Biotechnology Co., LTD. The experimental animals housed under a temperature of 22°C ~ 25°C, relative humidity of 50% ~ 60%, 12 hours of light and 12 hours of darkness. Adaptive feeding for 1 week. Rodent Diet with 60% fat Kcal% was purchased from Jiangsu Medicience, Ltd. (Jiangsu Province, China). Rosuvastatin Calcium was purchased from Shanghai Macklin Biochemical Technology Co., Ltd. (Shanghai, China).

The insulin-resistant mice were then randomly divided into HFD group (high-fat diet, 10 mice) and RSV group (high-fat diet +5 mg/kg/d rosuvastatin, 10 mice). Both phycocyanin and rosuvastatin were administered by gavage for 8 weeks.

### 2.2 Establishment of insulin resistance mouse model

The HFD group was fed the diet with 60% fat Kcal%. The weight and fasting blood sugar of the mice were measured once a week. Fasting blood glucose was measured by tail vein puncture in mice using a handheld glucose meter (Yuwell580, Jiangsu Yuyue Company, China). Before testing fasting blood sugar, the mice fasted for 12 hours. After 12 weeks of high-fat diet, oral glucose tolerance test was used to measure the blood glucose levels of mice at 0, 30, 60, 90, 120 and 180 min after gavage of glucose (2 g/Kg), and to analyze whether the mice in the high-fat diet group reached the index of insulin resistance (the area under the curve showed a significant increase, and the peak value of the curve was significantly increased; Blood glucose levels remained significantly higher than normal 2 hours later). If the target is reached, the model is successful.

Then the insulin resistant mice were randomly divided into HFD group (high-fat diet, n = 10) and RSV group (high-fat diet +5 mg/kg/d rosuvastatin, n = 10), which were given by gavage for 8 weeks.

### 2.3 Sample collection

After 8 weeks of drug treatment, the mice were anesthetized with bromoethanol after fasting for 12 hours, and blood samples were collected from the orbit and the mice were sacrificed. Fasting blood glucose and fasting insulin levels were measured directly. The digestive tract of mice was dissected longitudinally using a sterile scalpel, and the contents were removed and placed in frozen storage tubes (200-500 mg/sample), immediately snap-frozen in liquid nitrogen and stored at - 80°C.

### 2.4 Metagenome sequencing

#### 2.4.1 Metagenomic sequencing process

The bacterial genomic DNA was extracted by the kit and its quality was tested. Randomly breaking genomic DNA into fragments of 300 to 350 bp; Then add the base “A” to the 3 'end of the DNA fragment to convert the sticky end; Magnetic bead screening and purification of the target fragments; DNA splices were added at the end of the target fragment by PCR amplification. Constructing and detecting sequencing library; The sequencing library was combined with the sequencing chip by bridge PCR. Sequencing was performed using Illumina HiSeq or MiSeq computer based on fragment size.

#### 2.4.2 Metagenome sequencing data analysis

Clean data were obtained through preliminary statistics and optimization of the original sequencing data, and then genome assembly and predictive analysis were conducted through software. According to the predicted results, gene function annotation and species annotation, species and functional composition analysis, and comparative analysis of species and functional differences were carried out.

#### 2.4.3 Metabolomics profiling and data analysis

To identify metabolites that may play an active role in the relationship between gut microbiota, metabolites, and insulin resistance, we targeted a group of metabolites previously associated with human gut microbiota-host co-metabolism. First, metabolites in mouse intestinal contents were extracted, and then GC-MS and other instruments and professional software were used to analyze and sort out the data as required.

### 2.5 Data analysis

Statistical analysis was performed using GraphPad Prism statistical software (version 10.1). Numerical data are expressed as average ± SD values. Inter-group comparisons were performed using the independent sample t test, paired sample t test, one-way analysis of variance (ANOVA), or two-way ANOVA. P < 0.05 was considered statistically significant.

## 3 Results

### 3.1 Rosuvastatin improved insulin resistance

**Figure 1:**
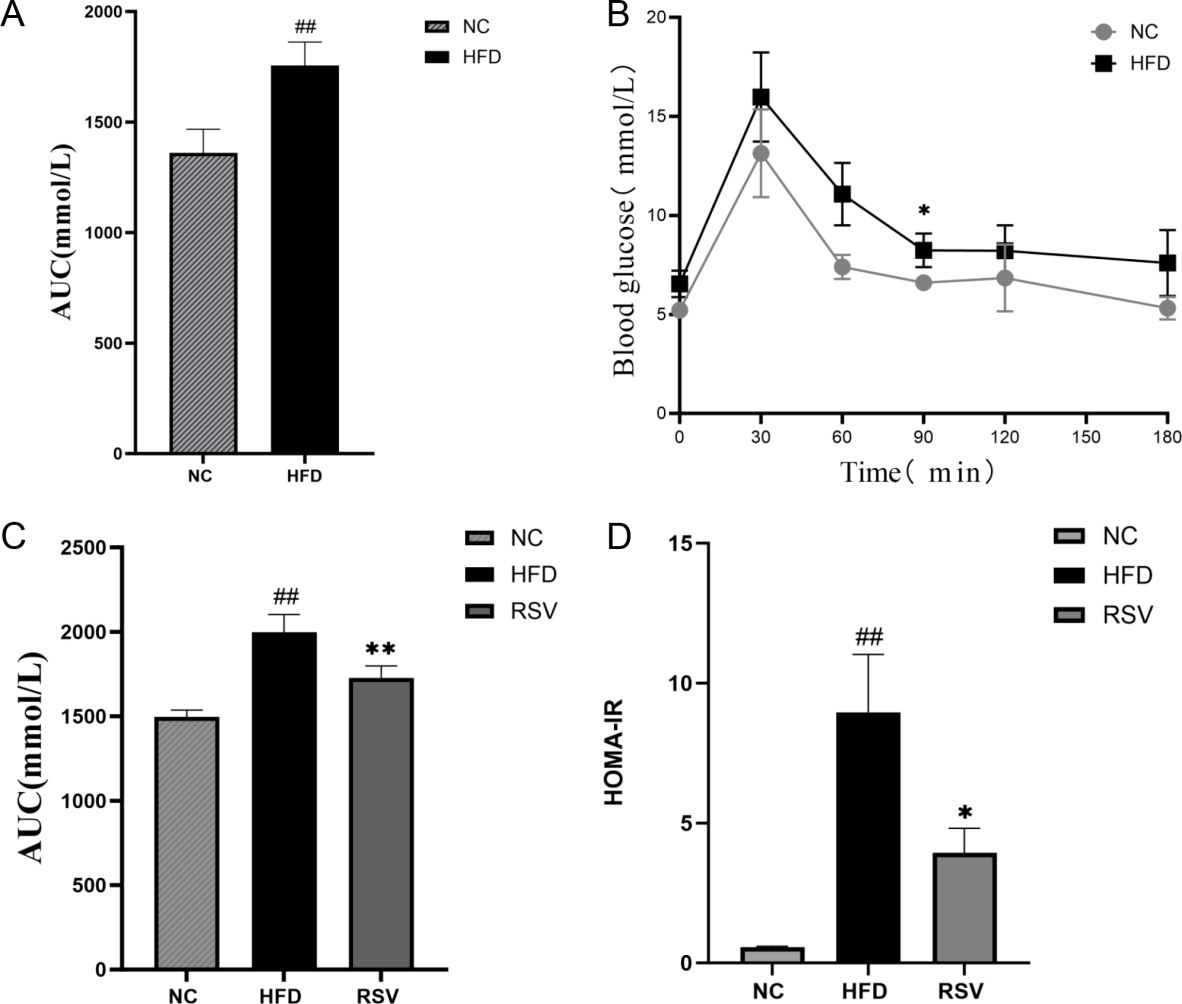
Oral rosuvastatin can repair impaired glucose tolerance. **(A)** After oral glucose (concentration of 2 g/KG) was measured at 0,30,60,90,120,150,180 minutes, it was found that 12 weeks of high-fat diet could caused a significant abnormal oral glucose tolerance curve of mice. **(B)** The area under the OGTT curve increased significantly due to the high fat diet. **(C)** The RVS group (that is, the HFD group of mice that took rosuvastatin orally for 8 weeks) tested by OGTT and found that rosuvastatin significantly reduced the area under the oral glucose tolerance curve. **(D)** HOMA-IR of RVS group mice decreased significantly after 8 weeks of oral administration of rosuvastatin. ##P <0.01 vs.NC, **P <0.01 vs. HFD, *P <0.05 vs. HFD, NC, normal control; HFD, high fat diet; RVS, high fat diet + 5mg/kg rosuvastatin.

First, the mice were fed a high-fat diet for 12 weeks, followed by an oral glucose tolerance test (OGTT) on an empty stomach. After gastric infusion of glucose (2 g/Kg), blood glucose levels were measured at 0, 30, 60, 90, 120 and 180 min, respectively. The results are shown in **Figure 1**, and compared with the NC group, the blood glucose levels of mice in the HFD group significantly increased at each time point. The area under the curve was still significantly higher in the HFD group than in the NC group. Thus, High fat diet can cause abnormal oral glucose tolerance in mice (**Figures 1A, B**), which can induce insulin resistance in mice.

Then, the insulin-resistant mice were treated with rosuvastatin for 8 weeks. Subsequently, the effects of rosuvastatin on fasting blood glucose and fasting insulin levels in these mice were measured. The result is shown in the figure (**Figures 1C, D**). The area under the curve of OGTT and HOMA-IR (Insulin Resistant Index) of insulin resistant mice induced by high-fat diet were still significantly higher than those of NC mice (1A). The opposite result was obtained after rosuvastatin administration after 8 weeks (1D). Thus, oral rosuvastatin can repair impaired glucose tolerance. These results suggest that rosuvastatin can improve insulin resistance.

### 3.2 Rosuvastatin induced changes in intestinal microbial composition of insulin-resistant mice

Next, in order to explore the mechanism of rosuvastatin in improving insulin resistance, we focused on the effect of rosuvastatin on gut microbial composition and its role. The intestinal contents of mice were tested by metagenomics. Firstly, the second generation sequencing Data quality statistics software cutadapt (v1.9.1) was used to remove adapters and low-quality sequences from the raw sequencing Data (Pass Filter Data) to obtain Clean Data for subsequent information analysis. The results are shown in **Table 1**.

**Table 1:** Clean data efficiency statistics.

Sample	#PF Reads	##Clean Reads	#Ratio of Reads(%)	#PF Bases(bp)	#Clean Bases(bp)	#Ratio of Bases(%)
HFD1	68388982	67878404	99.25	1.03E+10	1E+10	97.61
HFD2	73242900	67017544	91.5	1.1E+10	9.88E+09	89.94
HFD3	69217106	67647160	97.73	1.04E+10	1E+10	96.27
RSV1	81500082	68816780	84.44	1.22E+10	1.02E+10	83.23
RSV2	65602644	65316920	99.56	9.84E+09	9.69E+09	98.51
RSV3	64185702	63895424	99.55	9.63E+09	9.48E+09	98.43

**Figure 2:**
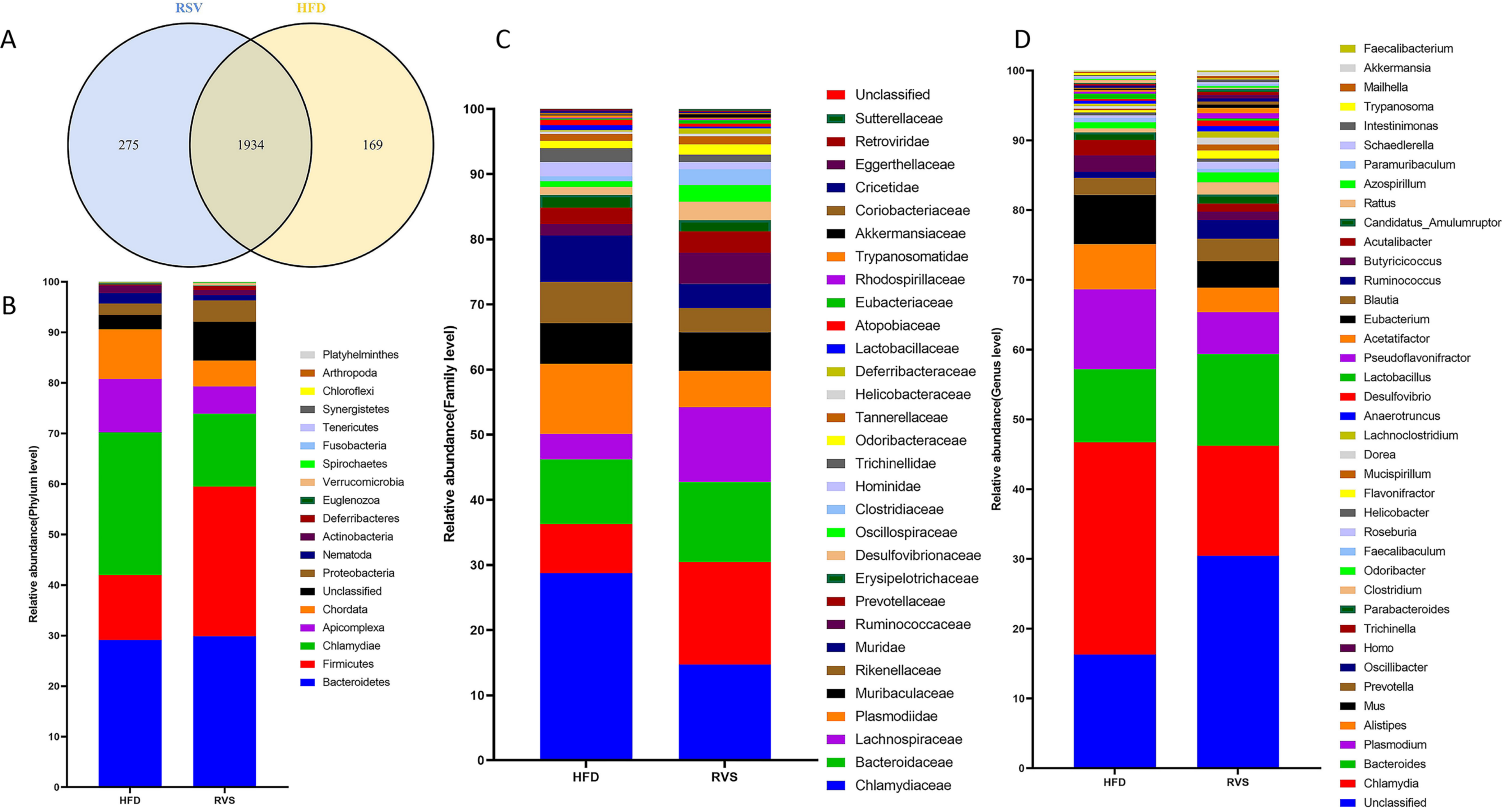
Changes in intestinal microbiome composition after rosuvastatin treatment. **(A)** Composition of gut microbes at genus level. **(B)** Differences in intestinal biota composition at phylum level. **(C)** Differences in intestinal biota composition at family level. **(D)** Differences in intestinal biota composition at genus level.

Subsequently, the insulin-resistant mice were treated with rosuvastatin, and the gut microbial composition was determined by metagenomic sequencing. As shown in the figure, the gut microbial composition changed significantly, with 2103 and 2209 genera found in the HFD and RSV groups, respectively (**Figure 2A**). Compared with HFD group, the relative abundance of Firmicutes, Proteobacteria and *Verrucomicrobia* in RSV group was significantly increased at the phylum level (263%, 216% and 484%, respectively). It was also found that the relative abundance of *Candidatus_Saccharibacteria* increased by 2727% after rosuvastatin treatment. The relative abundance of *Chlamydiae, Apicomplexa* and *Chordata* decreased significantly (by 172%, 170% and 169%, respectively) (**Figure 2B**). In the prediction results of family level, it was found that rosuvastatin could induce the relative abundance of *Akkermansiaceae*, *Deferribacteraceae*, *Lachnospiraceae*, *Ruminococcaceae*, *Oscillospiraceae*, *Desulfovibrionaceae*, *Bacteroidaceae* and *Clostridiaceae* are showing a remarkable increase (by 640%, 484%, 339%, 309%, 343%, 260%, 140%, and 360%, respectively). It was also found that rosuvastatin could increase the relative abundance of *Sutterellaceae* by 7380%. The relative abundance of *Lactobacillaceae, Chlamydiaceae, Plasmodiidae, Muridae, Trichinellidae* and *Rikenellaceae* decreased significantly (by 229%, 172%, 170%, 168%, 170% and 152%, respectively). (**Figure 2C**). The top 30 bacteria genera with high relative abundance in intestinal flora were analyzed. It was found that rosuvastatin can cause a significant increase in the relative abundance of *Oscillibacter, Clostridium, Roseburia, Flavonifractor, Faecalibacterium* and *Akkermansia* (An increase of 341%, 360%, 297%, 373%, 298% and 640%, respectively). However, the relative abundance of *Chlamydia, Plasmodium, Alistipes, Mus, Homo and Trichinella* decreased significantly (by 172%, 170%, 163%, 167%, 171% and 170%, respectively) (**Figure 2D**). In conclusion, rosuvastatin can cause changes in intestinal flora of insulin-resistant mice.

### 3.3 Gene function annotation was carried out in the metagenomic detection results of intestinal microbes in insulin-resistant mice after taking rosuvastatin

**Figure 3:**
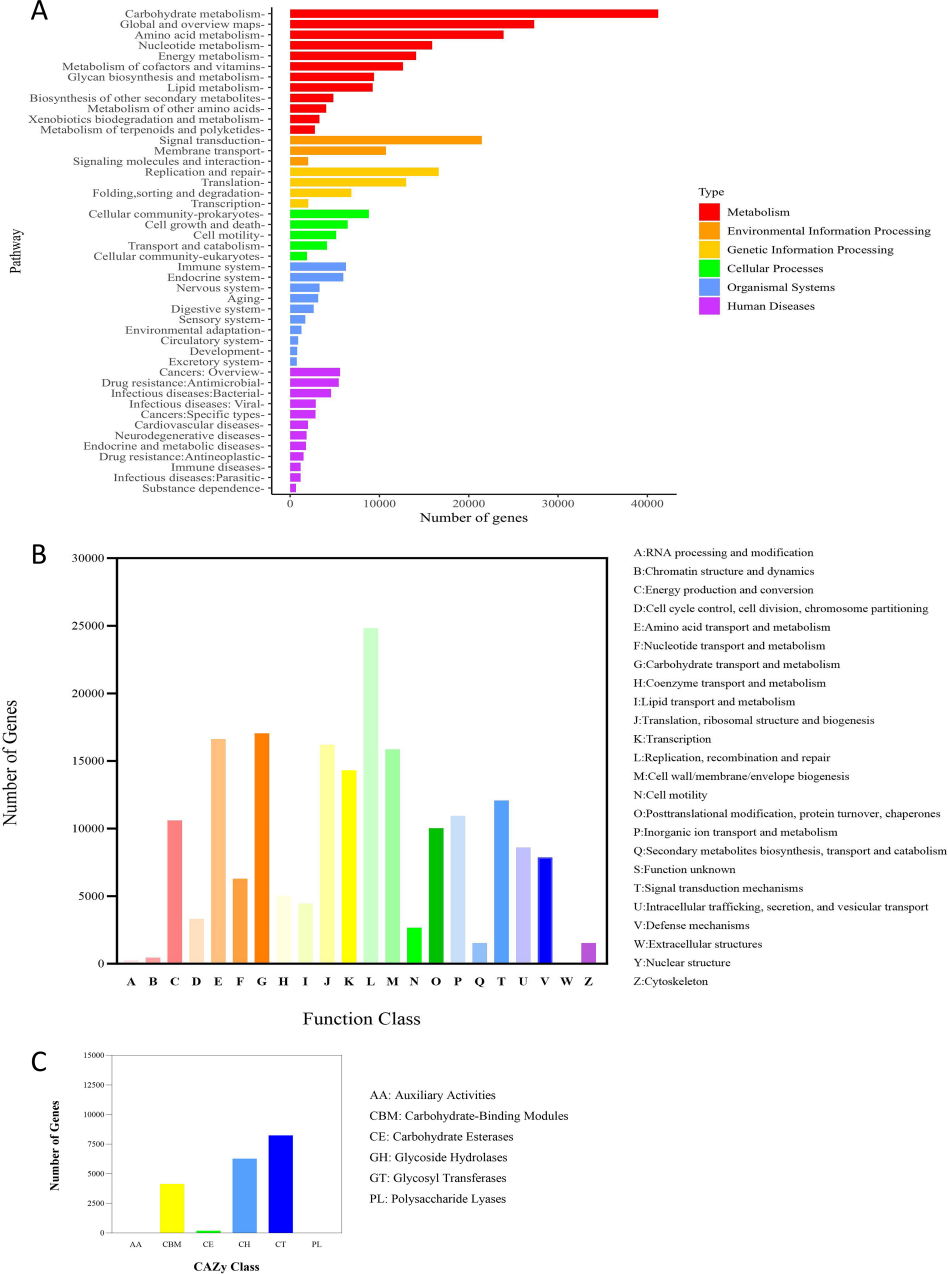
The number of genes in each pathway predicted by the metagenomic test results. **(A)** KEGG function annotation, **(B)** eggNOG function annotation, **(C)** CAZy function annotation.

After comparison with multiple databases, the genes of intestinal microbes in insulin-resistant mice treated with rosuvastatin were functionally annotated. According to the functional annotation of KEGG database, the number of genes enriched in Metabolism pathways was the largest among the 6 types of biological metabolic pathways, and the majority of genes in Metabolism pathways were enriched in Carbohydrate Metabolism pathways. In addition, among the Metabolism pathways, the number of genes enriched in Amino acid metabolism, Energy metabolism and Nucleotide metabolism was the second (**Figure 3A**). Gene function annotation through eggNOG database found that among the known functional classifications, the number of genes ranked in the top three were: L: Replication, recombination and repair, G: Carbohydrate transport and metabolism and E: Amino acid transport and metabolism (**Figure 3B**). Gene function annotation through CAZy database found that in 6 types of carbohydrate active enzymes, the top three gene numbers were Glycoside Hydrolases (GHs), Glycosyl Transferases (GTs) and Carbohydrate Binding Modules (CBMs) (**Figure 3C**). According to the above results, it could be inferred that the function of intestinal microbial genes is more inclined to the metabolic pathway, especially the carbohydrate metabolic pathway.

### 3.4 Effect of rosuvastatin administration on short-chain fatty acids in intestinal contents of mice

**Figure 4:**
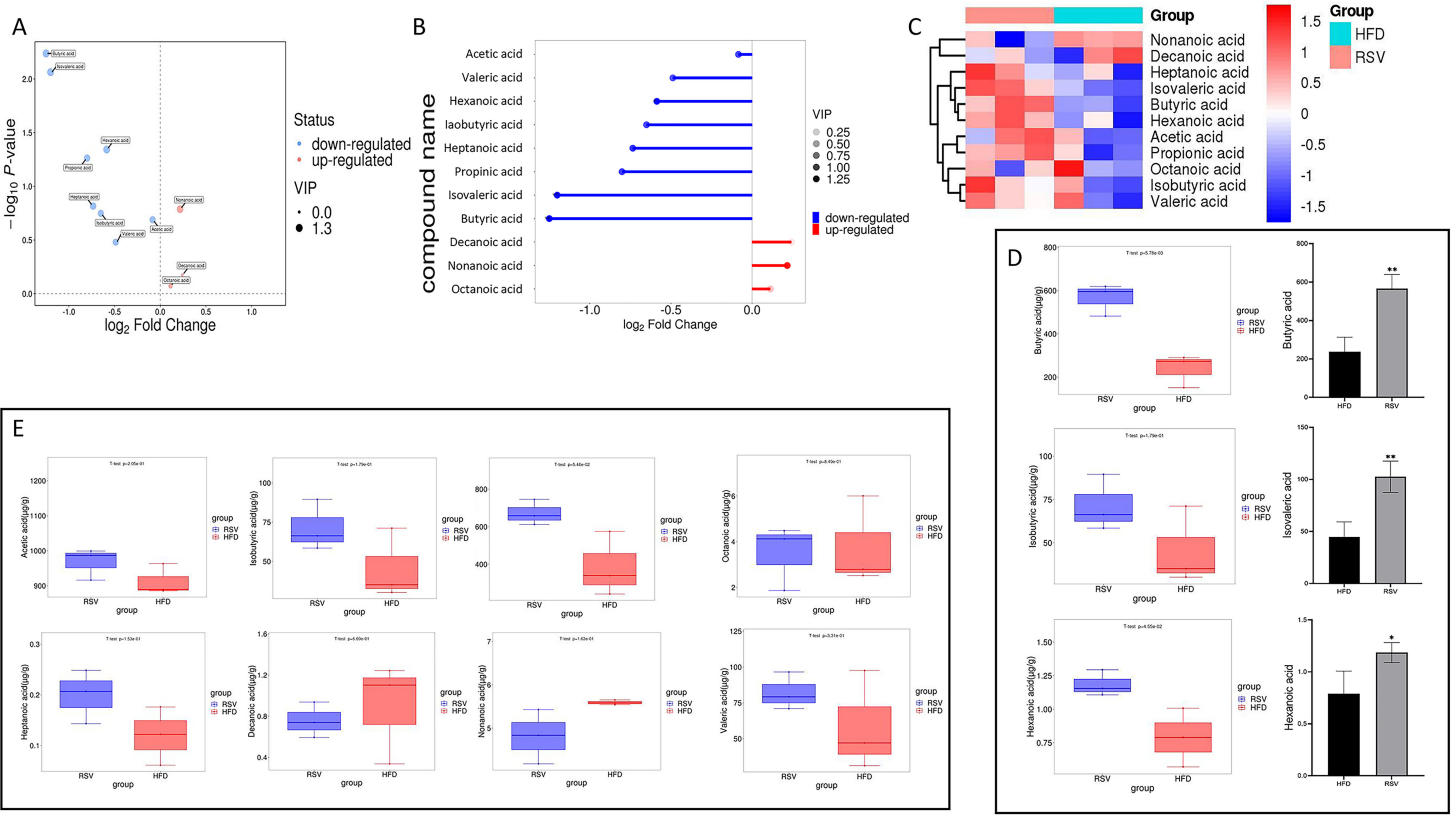
Effect of rosuvastatin administration on intestinal metabolites in insulin resistant mice. **(A–C)** Volcano, matchstick, and heat maps of the 11 fatty acids selected. **(D)** Boxplot of three significantly increased SCFAs; **(E)** 8 fatty acids that did not change significantly. *P <0.05, **P <0.01 vs. HFD group.

Target metabolomics analysis was performed by gas chromatography mass spectrometry (GC-MS) to identify the metabolites. After taking rosuvastatin for 8 weeks, 11 metabolites were detected in the intestinal contents of insulin-resistant mice, of which 3 SCFAs were decreased and 8 fatty acids were increased (**Figures 4A, B**). Among them, the contents of acetic acid, valeric acid, caproic acid, isobutyric acid, heptanoic acid, propionic acid, isovaleric acid and butyric acid increased, while the contents of caprylic acid, pelanoic acid and capric acid decreased (**Figures 4C-E**). In these changes, the contents of butyric acid, hexanoic acid and isovaleric acid were significantly increased (**Figure 4D**). Current studies suggested that an increased abundance of butyrate produced by gut bacteria in non-diabetic individuals was associated with a reduced incidence of type 2 diabetes and a reduced incidence of insulin resistance. These results suggested that rosuvastatin can affect the production of SCFAs in intestinal metabolites. This was consistent with the results of previous experiments, which showed that there were multiple increased bacterial populations that can produce butyric acid.

### 3.5 Butyric acid was a critical molecule in the intestinal metabolites of mice with high-fat diet-induced insulin resistance

In the metagenomic assay above, rosuvastatin administration was found to increase the abundance of multiple microflora in the gut of insulin-resistant mice. In the metagenomic assay above, rosuvastatin administration was found to increase the abundance of multiple microflora in the gut of insulin-resistant mice. The focus was on the significant increase of *Firmicutes* in the dominant bacteria after taking rosuvastatin. It was found that *Oscillibacter, Clostridium, Roseburia, Flavonifractor, Faecalibacterium* and *Akkermansia*, all of which belong to *Firmicutes*, have increased significantly. They were also surprised to find that all six bacteria could break down intestinal contents to produce a short-chain fatty acid, butyric acid. *Roseburia, Flavonifractor* and *Akkermansia* have been found to be negatively correlated with the occurrence of obesity or diabetes (Coppola et al., 2021; Baranova et al., 2024).

**Figure 5:**
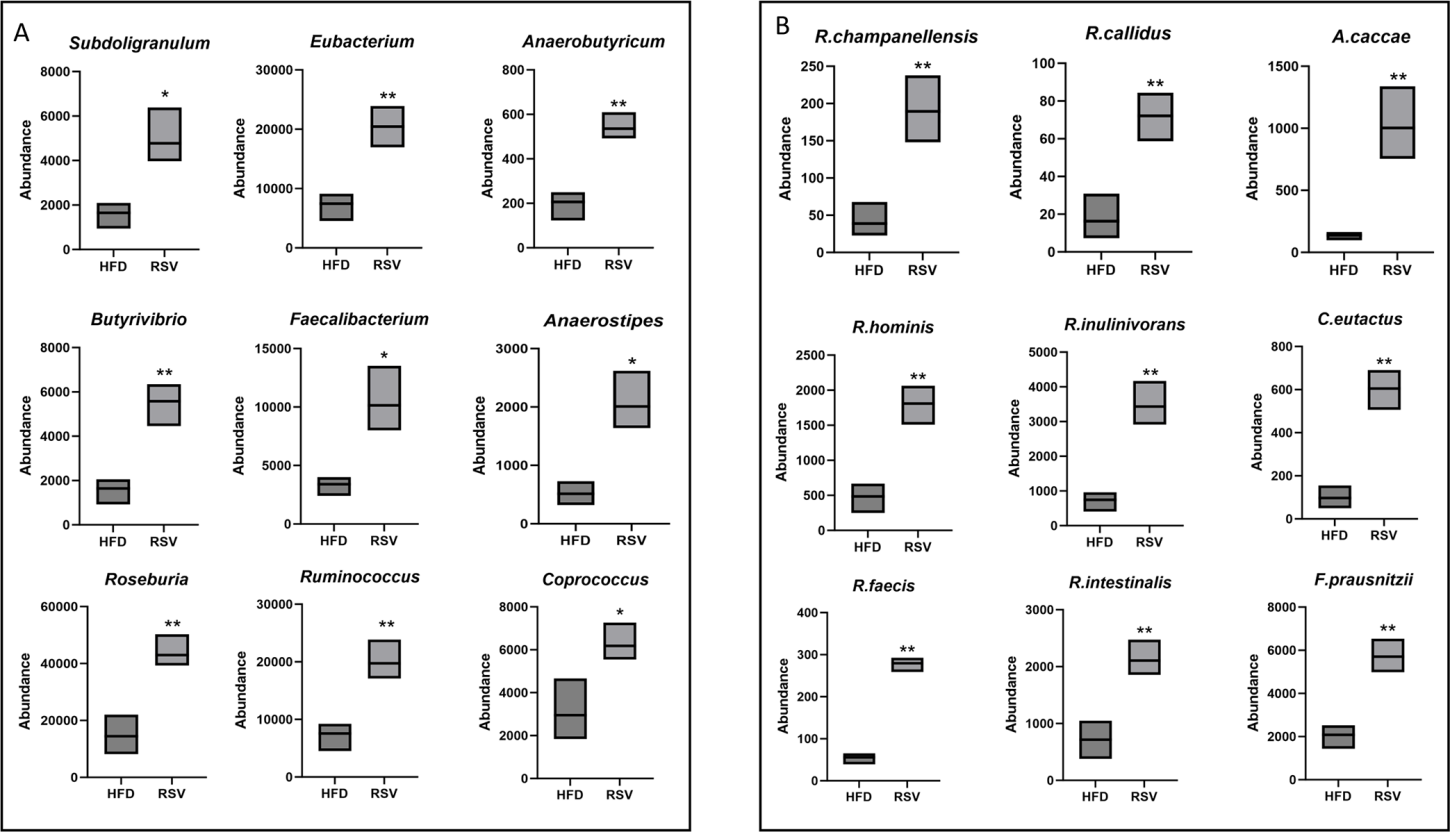
Significantly elevated bacterial flora associated with butyric acid production. **(A)** Genus that was significantly elevated and could in turn promote butyric acid production. **(B)** species that were significantly elevated and could promote butyrate biogenesis. *: P <0.05, **: P <0.01 vs. HFD group.

In addition to the bacteria genera in *Firmicutes* related to butyric acid production mentioned above, eight other dominant bacteria genera were found, including *Subdoligranulum, Anaerotruncus, Ruminococcus, Anaerostipes, Butyrivibrio, Eubacterium, Coprococcus* and *Anaerobutyricum*. The abundance of eight bacterial genera was statistically significant (**Figure 5A**). Of these genera, 19 species were involved in butyric acid anabolic processes, Such as *Faecalibacterium_prausnitzii, Subdoligranulum_variabile, Anaerotruncus_colihominis, Ruminococcus_bromii, Ruminococcus_champanellensis, Ruminococcus_callidus, Roseburia_inulinivorans, Roseburia_hominis, Roseburia_intestinalis, Roseburia_faecis, Anaerostipes_hadrus, Anaerostipes_caccae, Butyrivibrio_fibrisolvens, Eubacterium_rectale, Eubacterium_limosum, Eubacterium_rectale, Coprococcus_comes, Coprococcus_eutactus, Coprococcus_catus*. Among them, 9 bacterial species were statistically significant (**Figure 5B**).

**Figure 6:**
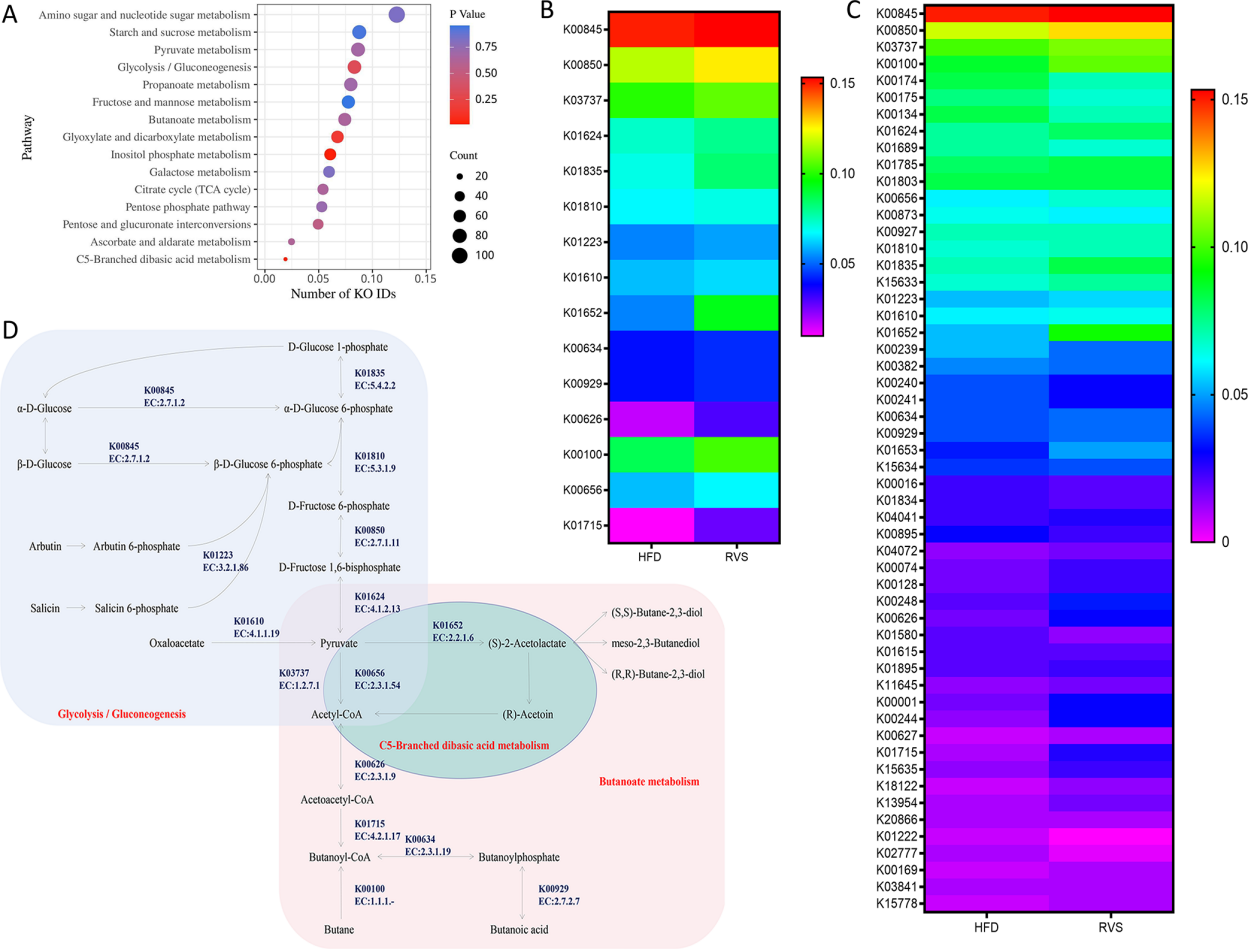
Administration of rosuvastatin mainly affected the metabolism of carbohydrates in intestinal metabolites of insulin resistant mice. **(A)** The major pathways by which rosuvastatin affects carbohydrate metabolism. **(B)** KO IDs involved in C5-Branched dibasic acid metabolism, Butanoate metabolism and Glycolysis/Gluconeogenesis. **(C)** Bacterial species underlying significant KO IDs. **(D)** Metabolic diagram.

Combined with the above analysis results, more attention was paid to the functions of metabolic pathways mainly focusing on carbohydrate metabolic pathways. The function and enrichment of carbohydrate metabolic pathways were analyzed by the metabolomics of intestinal contents in mice. Involved in Amino sugar and nucleotide sugar metabolism (109 KO IDs), Starch and sucrose metabolism (78 KO IDs), Pyruvate metabolism (77 KO IDs), Glycolysis/Gluconeogenesis (74 KO IDs), Propanoate metabolism (71 KO IDs), Fructose and mannose metabolism (69 KO IDs), Butanoate metabolism (66 KO IDs), Glyoxylate and dicarboxylate metabolism (60 KO IDs), Inositol phosphate metabolism (54 KO ids), Galactose metabolism (53 KO IDs), Citrate cycle (TCA cycle) (48 KO IDs), Pentose phosphate pathway (47 KO IDs), Pentose and glucuronate interconversions (44 KO IDs), Ascorbate and aldarate metabolism (22 KO IDs), C5-Branched dibasic acid metabolism (17 KO IDs). C5-Branched dibasic acid metabolism was significantly enriched in these metabolic pathways (p < 0.05) (**Figure 6A**).

Specific KO ID analysis was conducted by targeting C5-Branched dibasic acid metabolism, Butanoate metabolism and Glycolysis/Gluconeogenesis (**Figure 6C**). A total of 15 key KO IDs were selected. The key enzymes that essential for Acetyl-CoA formation are: glucokinase (K00845, EC:2.7.1.2), 6-phosphofructokinase 1 (K00850, EC:2.7.1.11), fructose-bisphosphate aldolase, class II (K01624, EC:4.1.2.13), phosphoglucomutase (K01835, EC:5.4.2.2), glucose-6-phosphate isomerase (K01810, EC:5.3.1.9), phosphoenolpyruvate carboxykinase (ATP) (K01610, EC:4.1.1.49), 6-phospho-beta-glucosidase (K01223, EC:3.2.1.86), pyruvate-ferredoxin/flavodoxin oxidoreductase (K03737, EC:1.2.7.1), which can thus affect butyric acid biosynthesis. Other enzymes play important roles in the formation of butyrate and butyrate by acetylCoa, such as butanol dehydrogenase (K00100, EC:1.1.1.-), acetolactate synthase I/II/III large subunit (K01652, EC:2.2.1.6), formate C-acetyltransferase (K00656, EC:2.3.1.54), butyrate kinase (K00929, EC:2.7.2.7), phosphate butyryltransferase (K00634, EC:2.3.1.19), acetyl-CoA C-acetyltransferase (K00626, EC:2.3.1.9), enoyl-CoA hydratase (K01715, EC:4.2.1.17) (**Figure 6B**).

Next, we focused on the relationship between 15 key enzymes in the carbohydrate metabolism pathway and intestinal microorganisms in promoting butyric acid metabolism and drew the following figure (**Figure 6D**).

## 4 Discussion

In this study, insulin-resistant mice were treated with rosuvastatin for 8 weeks, resulting in a significant improvement in insulin resistance. Following this treatment, metagenomic sequencing and metabolomic analysis were conducted on the intestinal contents of the mice. The aim was to investigate the effects of rosuvastatin on intestinal microorganisms and metabolites in insulin-resistant mice, as well as to explore the relationship between rosuvastatin and the improvement of insulin resistance. The results showed that rosuvastatin increased the diversity of intestinal flora in mice, and also changed the abundance of many microbial communities. Among the flora with altered abundance, it was found that most functions were concentrated in metabolic pathways, many of which could promote the production of butyric acid, a metabolite found in the gut. Furthermore, the analysis of metabolomics results from the intestinal contents of mice revealed that rosuvastatin could alter the levels of various short-chain fatty acids. Notably, the amounts of butyric acid, caproic acid, and isovaleric acid were significantly increased. Research has shown that the content of butyric acid is negatively correlated with obesity and diabetes. However, the connections between caproic acid and isovaleric acid levels and insulin resistance remain unclear and would be investigated in the next study. We focused on 19 bacterial species and 15 key enzymes that involved in butyric acid production and carbohydrate metabolism. Therefore, we speculated that rosuvastatin might improve insulin resistance by increasing the abundance and metabolite content of intestinal microbiota and affecting the overall carbohydrate metabolism.

The gut microbiota is formed at birth and changes as we age. It exists in a commensal relationship with the host and is an essential component of the human body. This microbiota comprises hundreds of different species of bacteria, which are primarily categorized into nine phyla. It is primarily composed of the phyla *Firmicutes, Bacteroidetes, Proteobacteria, Actinobacteria*, and *Fusobacteria*, which together account for 90% of the total human microbiota. Research has shown that a long-term high-fat diet can significantly alter the structure of intestinal flora (Zhang et al., 2020; Zhu et al., 2024). Changes in the composition of intestinal microbes are linked to HOMA-IR and type 2 diabetes (Alvarez-Silva et al., 2021; Chen et al., 2021). For instance, the presence of bacteria from families such as *Christensenellaceae, Marvinbryantia*, and certain unclassified *Ruminococcaceae* is negatively correlated with HOMA-IR levels. Conversely, a high abundance of *Clostridiaceae, Peptostreptococcaceae, Clostridium sensu stricto, Intestinibacter* or *Romboutsia* is associated with a lower incidence of type 2 diabetes. These bacteria are all known to produce butyrate. In this study, administration of rosuvastatin significantly increased the abundance of the aforementioned butyrate-producing bacteria in the intestinal tract of mice. SCFAs are carboxylic acids generated from the fermentation of dietary fiber and resistant starch in the cecum and colon by gut bacteria (Koh et al., 2016; Ratajczak et al., 2019). The primary SCFAs include acetate (C2), propionate (C3), and butyrate (C4). SCFAs are primarily produced by the *Firmicutes*, some of which are transported to the colonic epithelium to supply energy for their production (Zhang et al., 2023). The remaining SCFAs are released from the intestine and enter the circulatory system via the liver and portal circulation. In this study, following treatment with rosuvastatin, a significant increase was observed in the intestinal abundance of *Firmicutes*, as well as a notable rise in the levels of butyric acid, a four-carbon compound.

In this study, insulin-resistant mice taking rosuvastatin also caused changes in the gut microbiota and its metabolites. It is speculated that these changes may be related to the improvement of insulin resistance by rosuvastatin. Research has found that obesity-related microbiome dysregulation was inversely associated with statin therapy (Vieira-Silva et al., 2020). In the study conducted by Jiyeon Kim, the administration of atorvastatin and rosuvastatin resulted in a substantial increase in the abundance of *Bacteroides, Butyricimonas*, and *Mucispirillum* (Kim et al., 2019). In our research, we observed that the administration of rosuvastatin to insulin-resistant mice resulted in a notable increase in the prevalence of *Firmicutes, Proteobacteria*, and *Verrucomicrobia* within the gastrointestinal tract. In the phylum *Firmicutes*, the genera *Oscillibacter, Clostridium, Roseburia, Flavonifractor, Faecalibacterium*, and *Akkermansia* have demonstrated a significant increase in abundance and possess the capability to produce butyric acid. Additionally, this analysis encompassed 15 critical enzymes that are integral to carbohydrate metabolism and are related to the production of butyric acid. In this study, the administration of rosuvastatin resulted in a significant increase in butyrate production in insulin-resistant mice. Butyrate serves as the primary source of energy for colonic epithelial cells, and a substantial portion of the butyrate that is absorbed may have already been utilized by the colonic mucosa. Intestinal butyric acid plays a significant role in the prevention and management of obesity and insulin resistance through the activation of G-protein-coupled receptors (GPCRs) (Kim et al., 2013; Kim et al., 2014; D'Souza et al., 2017; Wang et al., 2024) and the inhibition of histone deacetylase (HDAC) (Davie, 2003; Steliou et al., 2012; Chriett et al., 2017; Wang et al., 2024). Although the expression of GPCRs and HDAC was not examined in this study, a significant improvement in insulin resistance was observed. Furthermore, butyrate supplementation has demonstrated various metabolic advantages, including the prevention of obesity and obesity-related diseases associated with a high-fat diet, as evidenced by animal model studies (Arnoldussen et al., 2017; Matheus et al., 2017; Pelgrim et al., 2017; Fang et al., 2019). A study conducted in mice has confirmed that butyrate played a significant role in regulating thermogenesis and energy homeostasis by activating lysine-specific demethylase (LSD). As a histone demethylase, LSD serves as a crucial regulator of thermogenesis, promoting thermogenic activity in brown adipose tissue (BAT) (Wang et al., 2020). The alterations in intestinal microbiota and the observed increase in butyric acid production indicate that rosuvastatin significantly contributes to the improvement of insulin resistance in the murine model utilized in this study.

## Data Availability

Original datasets are available in a publicly accessible repository: The original contributions presented in the study are publicly available. This data can be found here: [https://www.ebi.ac.uk/ena/browser/view/PRJEB90470, accession number PRJEB90470].
